# Supplementary figures and images for: Understanding the biogeographic processes behind the accumulation of modern-day marine biodiversity
Source: bioRxiv. 2026 Jan 9:2026.01.09.697399. Preprint. [Version 1] doi: 10.64898/2026.01.09.697399 (PMC12803097; doi:10.64898/2026.01.09.697399)

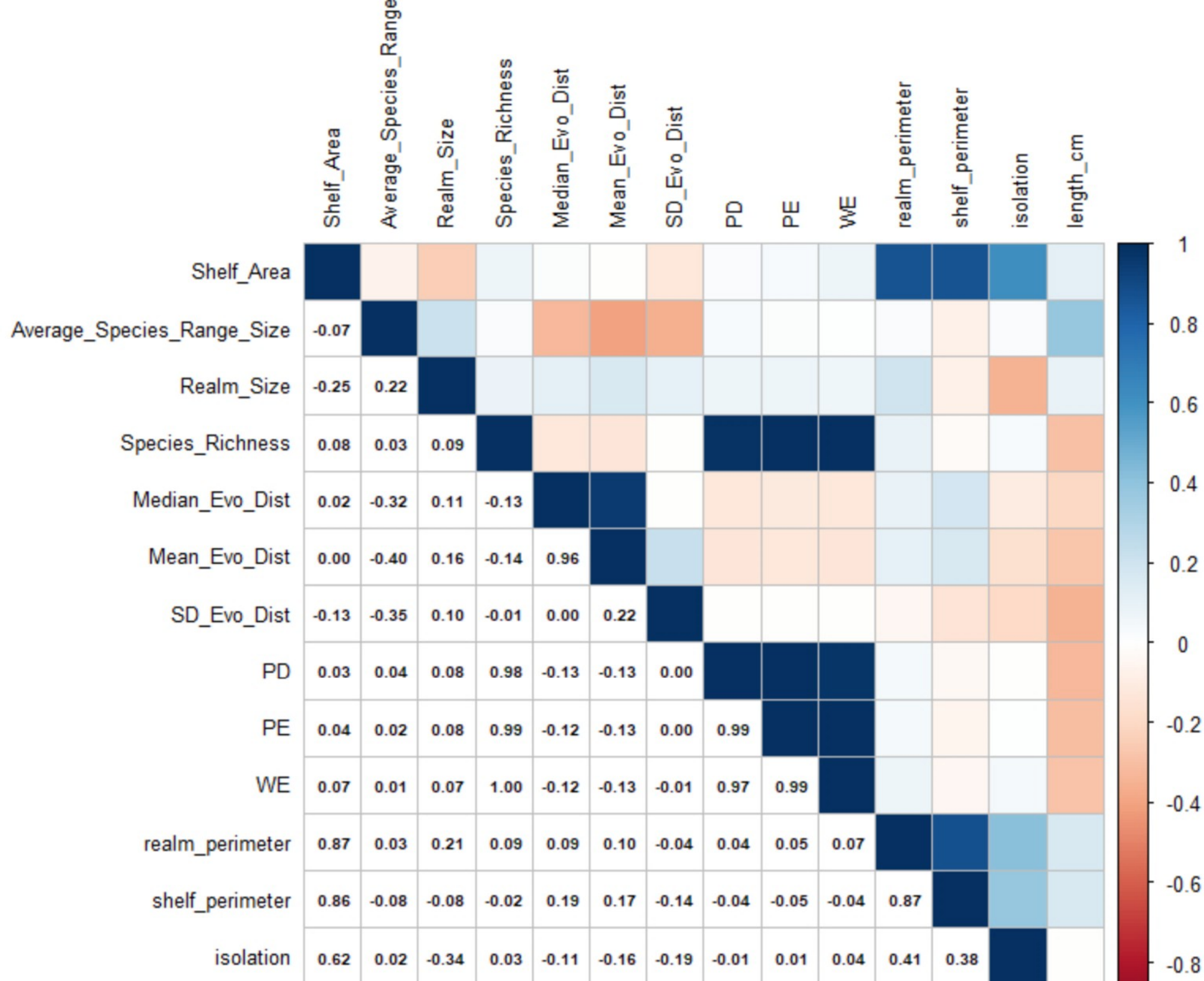

Supplement: Supplement 2 [file media-2.pdf]
